# Pre-Emptive Upregulation of Antimicrobial Peptides by Dietary Propolis Improves Ethanol Tolerance in *Drosophila melanogaster*

**DOI:** 10.3390/insects17060542

**Published:** 2026-05-22

**Authors:** JooHeon Cha, Young Ho Kim

**Affiliations:** 1 Department of Vector Entomology, Kyungpook National University, Sangju 37224, Republic of Korea; 19cjh95@gmail.com; 2 Research Institute of Invertebrate Vector, Kyungpook National University, Sangju 37224, Republic of Korea

**Keywords:** antimicrobial peptides, ethanol tolerance, IMD pathway, chemical stress, *Drosophila melanogaster*, propolis

## Abstract

Ethanol is a pervasive chemical stressor in fermentative environments and represents a major ecological challenge for *Drosophila melanogaster*, a species that naturally inhabits decaying fruits. Although ethanol tolerance has traditionally been attributed to detoxification and antioxidant pathways, accumulating evidence indicates that immune-related genes, particularly those encoding immune deficiency (IMD) pathway-associated antimicrobial peptides (IMD-AMPs), contribute importantly to chemical stress adaptation. Previous studies have demonstrated that IMD-AMP induction is required for ethanol tolerance; however, whether elevated IMD-AMP expression alone is sufficient to enhance tolerance has remained unresolved. In this study, we investigated the functional significance of IMD-AMP upregulation in ethanol tolerance using dietary propolis as an experimental immune-modulating agent. *D. melanogaster* were reared throughout their life cycle on propolis-supplemented diets and subsequently exposed to ethanol. Propolis-fed flies exhibited significantly enhanced survival under ethanol stress compared with control flies. Notably, this increased tolerance was not accompanied by upregulation of classical ethanol metabolism genes or broad induction of antioxidant-related genes. Instead, propolis feeding increased baseline and early-stage expression of IMD-AMP genes, including *Diptericin A* (*DptA*), *Diptericin B* (*DptB*), *Attacin* (*AttC*), and *Metchnikowin* (*Mtk*) before and during ethanol exposure. These findings suggest IMD-AMP upregulation is positively associated with enhanced ethanol tolerance in *D. melanogaster*. Our results establish a proactive role for immune-related pathways in chemical stress resistance and extend the functional scope of AMPs beyond pathogen defense. This study identifies IMD-AMPs as key effectors linking immune activation to physiological adaptation under ethanol-induced chemical stress.

## Simple Summary

The common fruit fly, *Drosophila melanogaster*, naturally inhabits fermenting fruits that contain ethanol, a substance that can be toxic. While scientists have typically focused on how flies break down ethanol, recent evidence suggests that the immune system also helps them survive this challenge. In this study, we investigated whether increasing antimicrobial peptide expression could enhance ethanol tolerance. Flies were fed propolis—a natural substance collected by honeybees—to boost immune defenses. Flies reared on a propolis-supplemented diet survived ethanol exposure much better than those on a standard diet. Notably, this enhanced survival was not associated with increased ethanol metabolism or typical antioxidant genes. Instead, propolis-fed flies exhibited elevated expression of antimicrobial peptide genes associated with the immune deficiency pathway and improved survival under ethanol stress. These findings demonstrate that diet-induced immune priming can directly improve ethanol tolerance. Understanding the connection between immunity and chemical stress provides insight into how insects adapt to changing environments and how nutrition helps them survive chemical threats in nature.

## 1. Introduction

Insects are continuously exposed to diverse chemical stressors, including plant secondary metabolites, microbial fermentation products, and anthropogenic xenobiotics, in their habitats. To cope with these challenges, insects have evolved complex physiological and molecular strategies that collectively determine chemical tolerance and survival. Traditionally, detoxification enzymes involved in xenobiotic metabolism, such as alcohol dehydrogenases, cytochrome P450 monooxygenases, and antioxidant-related enzymes, have been regarded as the primary mediators of chemical tolerance [1,2]. However, increasing evidence indicates that these classical pathways alone cannot fully explain the variation in chemical stress resistance observed among insect species or populations adapted to different ecological conditions.

Among the environmental chemicals encountered by insects, ethanol is a particularly relevant and recurrent stressor for *Drosophila melanogaster*. *Drosophila melanogaster* predominantly inhabits decaying and fermenting fruits, where microbial activity—primarily driven by yeasts—continuously converts sugars into ethanol [3,4]. Consequently, *D. melanogaster* is chronically exposed to ethanol throughout its life cycle, often at concentrations that impose considerable physiological and oxidative stress [3,5]. Therefore, ethanol functions not only as a chemical cue influencing habitat selection and oviposition behavior but also as a persistent toxic stressor that shapes survival and physiological adaptation [6,7]. Comparative ecological studies further highlight the role of ethanol in shaping chemical tolerance in *D. melanogaster*. Unlike *Drosophila suzukii*, which preferentially infests fresh, intact fruits with minimal fermentation, *D. melanogaster* exhibits markedly higher tolerance to ethanol and other fermentation-associated chemicals [7,8]. Experimental comparisons between the two species have demonstrated that *D. melanogaster* survives much better under ethanol exposure, reflecting long-term adaptation to fermentative environments. This interspecific difference underscores the physiological specialization of *D. melanogaster* for chemical stressors associated with fruit decay and fermentation and suggests that ethanol tolerance is a defining trait of its ecological niche [7].

Ethanol tolerance has traditionally been attributed to metabolic detoxification pathways, particularly those involving alcohol dehydrogenase (Adh) and aldehyde dehydrogenase (Aldh), as well as antioxidant-related enzymes that mitigate ethanol-induced oxidative stress [1]. However, comparative and functional studies have demonstrated that differences in ethanol tolerance cannot be fully explained by variations in these metabolic or antioxidant pathways alone. Instead, ethanol exposure elicits a strong transcriptional response involving innate immune genes, especially those encoding antimicrobial peptides (AMPs). Transcriptomic analyses have revealed that ethanol exposure consistently induces robust upregulation of AMP genes in *D. melanogaster*, while classical detoxification genes often exhibit limited or variable responses. In particular, AMPs regulated by the immune deficiency (IMD) signaling pathway, including *Diptericin* (*Dpt*), *Attacin* (*AttC*), and *Metchnikowin* (*Mtk*), are among the most strongly induced genes following ethanol challenge [8]. Functional studies have further demonstrated that RNA interference (RNAi)-mediated suppression of the IMD pathway through *Relish* knockdown reduces AMP expression and significantly decreases survival under ethanol stress, establishing that IMD-dependent AMP (IMD-AMP) expression is required for ethanol tolerance [9]. Together, these findings support a close association between IMD-AMP expression and ethanol tolerance. However, an important mechanistic gap remains unresolved. Previous studies have primarily demonstrated that IMD-AMP induction occurs after ethanol exposure and that reduced IMD-AMP expression compromises survival under ethanol stress [8,9]. Although these results establish IMD-AMPs as necessary components of ethanol tolerance, they do not address whether increased IMD-AMP expression is sufficient to enhance ethanol tolerance. In other words, it is unclear whether increasing IMD-AMP expression before chemical exposure can actively improve ethanol tolerance rather than merely reflect a downstream stress response. Addressing this question requires an experimental framework capable of inducing sustained or pre-emptive upregulation of IMD-AMPs in the absence of acute chemical exposure. Dietary modulation provides a powerful approach to probe this possibility because chronic dietary intake can shape baseline physiological and immune states, thereby influencing organismal responses to subsequent stressors [10]. Propolis, a resinous mixture rich in plant-derived polyphenols and flavonoids, possesses well-documented immunomodulatory properties and has been shown to induce immune-related gene expression, including AMPs, in insects [11]. Although *D. melanogaster* is unlikely to encounter propolis directly in its natural environment, propolis serves as a chemically complex and biologically relevant experimental tool to induce immune activation and examine the functional consequences of elevated AMP expression. Importantly, dietary supplementation with propolis allows direct testing of whether sustained, diet-induced AMP upregulation can enhance ethanol tolerance, thereby extending beyond correlative observations toward functional validation. In this context, propolis exposure is not intended to mimic a natural feeding scenario but rather to experimentally induce an immune-primed state characterized by elevated IMD-AMP expression before ethanol challenge.

Based on the previously observed induction of IMD-AMP genes during ethanol exposure and the reduced tolerance following RNAi-mediated suppression of AMP expression [8,9], we hypothesized that pre-existing elevation of IMD-AMP expression induced by dietary propolis supplementation enhances ethanol tolerance in *D. melanogaster*. To test this hypothesis, flies were reared on a propolis-supplemented diet throughout their life cycle and subsequently exposed to ethanol as a model chemical stressor. Survival under ethanol exposure was assessed, and transcriptional responses of genes involved in alcohol metabolism, oxidative stress response, and innate immunity were quantified using quantitative real-time PCR (qRT-PCR). By integrating survival assays with targeted gene expression analyses, this study aimed to determine whether diet-induced IMD-AMP upregulation is sufficient to enhance ethanol tolerance independently of classical detoxification or antioxidant pathways.

By directly testing whether pre-existing elevation of IMD-AMP expression confers increased ethanol tolerance, this study completes a mechanistic framework linking IMD-AMPs to chemical stress adaptation. These findings provide functional evidence that immune activation is not merely a consequence of ethanol exposure but may also serve as a proactive determinant of chemical tolerance in insects.

## 2. Materials and Methods

### 2.1. Insect Rearing and Dietary Propolis Supplementation

*D. melanogaster* (Canton-S) was obtained from the Bloomington Drosophila Stock Center (Indiana University, Bloomington, IN, USA) and maintained under controlled laboratory conditions at 25 ± 2°C with a 16:8 h light:dark photoperiod and 45–55% relative humidity. Flies were reared on a standard artificial diet consisting of 1 L distilled water, 7.7 g agar (Duksan, Ansan, Republic of Korea), 40.8 g cornmeal (Hansol tech, Seoul, Republic of Korea), 84 g dextrose (Samchun chemicals, Seoul, Republic of Korea), 5 mL honeydew (Ever miracle, Jeonju, Republic of Korea), 62.5 g dried yeast (Duksan, Ansan, Republic of Korea), 14.6 mL mold inhibitor [Propionic acid (Alfa Aesar, Ward Hill, MA, USA) and Methyl 4-Hydroxybenzoate (Daejung Chemicals & Metals Co., Ltd., Siheung, Republic of Korea)], and 5.7 mL antibiotics [Oxytetracycline hydrochloride (DaOne OTC 50%; DaOne Chemical Co., Ltd., Siheung, Republic of Korea)] [12,13]. To examine the effects of chronic dietary exposure to propolis, flies were reared on an artificial diet supplemented with 1 g of propolis (Chong Kun Dang Pharmaceutical Corp., Seoul, Republic of Korea) throughout their life cycle. Control flies were maintained on the same diet without propolis supplementation. For the experiments, parental flies reared on the propolis-free diet were allowed to oviposit directly onto either the control or propolis-supplemented diet. Offspring remained on their respective diets from embryonic development through adulthood, ensuring continuous dietary exposure throughout the life cycle.

Adult female flies were used in all experiments to minimize variability associated with sex-specific differences in ethanol tolerance and gene expression. Newly eclosed flies were collected within 24 h of emergence and maintained with males for 24 h to allow mating. Mated females were separated and maintained on their respective diets for an additional 3–5 days before ethanol exposure assays. Mated females were used to minimize physiological variation associated with reproductive status and to standardize the adult population throughout the experiments.

### 2.2. Ethanol Tolerance Assay

To assess ethanol tolerance, adult female flies reared on a control or propolis-supplemented diet were exposed to ethanol (Supelco, Merck KGaA, Darmstadt, Germany) vapor. For each treatment and ethanol concentration, three biological replicate vials were prepared, each containing 10 adult female flies in 5 mL transparent glass vials sealed with gauze-covered caps to prevent direct contact with liquid ethanol while allowing vapor diffusion. A cotton plug placed inside each vial cap was soaked with 200 µL ethanol at the designated concentration. Vials were inverted to ensure uniform distribution of ethanol vapor. Ethanol concentrations (0, 5, 7.5, 10, 12.5, 15, 17.5, and 20%) were selected based on preliminary survival assays. For subsequent gene expression analyses, 12.5% (*v*/*v*) ethanol was used because this concentration produced a clear difference in survival between propolis-treated and control flies. Ethanol exposure was maintained for up to 8 h, with fresh ethanol added every 2 h to sustain vapor concentration. Survival was monitored at 1-h intervals. Flies exhibiting any movement, including antennal or leg twitching, were considered alive.

### 2.3. Sample Collection for Gene Expression Analysis

Based on ethanol tolerance results, surviving flies were collected for gene expression analysis at 0, 1, 2, 4, and 6 h after ethanol exposure. At each time point, flies were anesthetized with CO_2_ and transferred to sterile microcentrifuge tubes. Three surviving flies were pooled per biological replicate to reduce individual variability. All experiments were performed in triplicate. Samples were immediately frozen in liquid nitrogen and stored at −80°C until RNA extraction.

### 2.4. RNA Extraction and cDNA Synthesis

Total RNA was extracted using TRI Reagent^®^ (Zymo Research, Irvine, CA, USA). Frozen samples were homogenized in 200 µL TRI Reagent^®^ with three 2.8 mm stainless steel beads using an automated homogenizer (Bertin Technologies, Montigny-le-Bretonneux, France). Homogenization was performed for 70 s (30 s homogenization, 10 s pause, and 30 s homogenization) at 6500 rpm. RNA purification was conducted using the Direct-zol™ RNA Miniprep Plus Kit (Zymo Research) according to the manufacturer’s instructions, including on-column DNase I treatment to remove genomic DNA contamination. RNA concentration and purity were assessed spectrophotometrically.

First-strand cDNA was synthesized from 1 µg total RNA using oligo(dT) primers and ReverTraAce reverse transcriptase (Toyobo, Osaka, Japan) following the manufacturer’s protocol.

### 2.5. Quantitative qRT-PCR

qRT-PCR was performed using a CFX Connect qRT-PCR Detection System (Bio-Rad, Hercules, CA, USA) with SYBR Green chemistry. Each 20 µL reaction contained 10 µL 2× Thunderbird SYBR qPCR Master Mix (Toyobo), 1 µL each of forward and reverse primers (10 pmol), 5 µL diluted cDNA template (10 ng µL^−1^), and 4 µL nuclease-free water.

PCR conditions consisted of an initial denaturation at 95 °C for 1 min, followed by 40 cycles of 95 °C for 15 s, 58 °C for 15 s, and 72 °C for 30 s. All reactions were performed in triplicate.

Target genes included AMPs associated with the IMD pathway (*DptA*, *DptB*, *AttC*, and *Mtk*) and genes involved in alcohol metabolism and oxidative stress response. Ribosomal protein L18 (*RpL18*) was used as the reference gene for normalization. Primer sequences are provided in Table S1 [9,14].

### 2.6. Statistical Analysis

Survival and gene expression data were analyzed using SPSS software (version 29.0; IBM Corp., Armonk, NY, USA). Differences between control and propolis-treated groups were assessed using a generalized linear model (GLM) repeated-measures ANOVA. Gene expression levels were calculated using the ΔΔCt method, and results are presented as mean ± standard error of the mean (SEM). Statistical significance was defined at *p* < 0.05.

## 3. Results

### 3.1. Lifelong Dietary Propolis Supplementation Enhances Ethanol Tolerance

**Figure 1:**
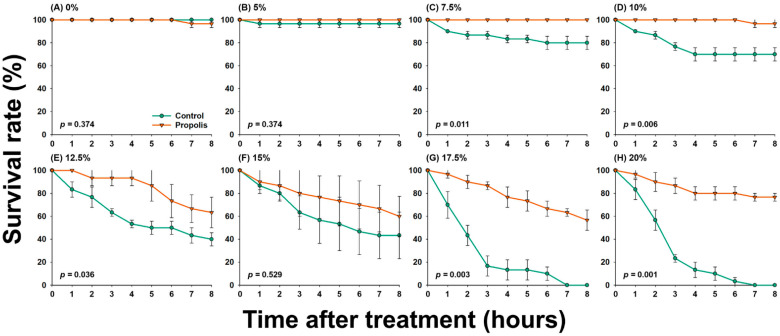
Ethanol tolerance of *D. melanogaster* reared on a propolis-supplemented diet. Survival of adult female *D. melanogaster* reared throughout development on a control or propolis-supplemented diet following ethanol exposure. Flies were exposed to ethanol concentrations ranging from 0% to 20% (*v*/*v*), and survival was monitored hourly for 8 h. Propolis-fed flies exhibited significantly higher survival than control flies at moderate ethanol concentrations, including at 12.5%, which was selected for subsequent gene expression analyses. Data represent mean survival percentages from three biological replicates. Data are presented as mean ± SEM. Statistical analysis was performed using GLM repeated-measures ANOVA (*p* < 0.05).

Across ethanol concentrations ranging from 5% to 20% (*v*/*v*), propolis-treated flies exhibited enhanced tolerance compared with control flies (Figure 1). At low ethanol concentrations (0–5%), both groups showed nearly complete survival, indicating no baseline difference in viability (Figure 1A,B). However, survival trends diverged as ethanol concentration increased. From 7.5% ethanol onward, propolis-treated flies generally maintained higher survival than control flies, except at 15% ethanol, where no significant difference was detected. At moderate ethanol concentrations (7.5–12.5%), propolis-treated flies exhibited a clear survival advantage, whereas survival in control flies progressively declined over time (Figure 1C–F). At 12.5% ethanol, which was selected for subsequent gene expression analyses, survival at the 8 h time point was significantly higher in the propolis-treated group (63.33%) than in the control group (40.0%; *p* = 0.036) (Figure 1E). At higher ethanol concentrations (≥17.5%), survival in the control group declined rapidly, reaching complete mortality before the end of the exposure period, whereas some propolis-treated flies survived longer (Figure 1G,H). At 20% ethanol, mortality in the propolis-treated group occurred primarily during the early exposure period, after which survival remained relatively stable, likely reflecting the persistence of a subset of highly ethanol-tolerant individuals (Figure 1H).

Overall, these results demonstrate that lifelong dietary propolis supplementation enhances ethanol tolerance in *D. melanogaster*, establishing a robust phenotypic basis for subsequent analyses of the underlying molecular mechanisms.

### 3.2. Propolis Does Not Enhance Ethanol Tolerance via Upregulation of Alcohol Metabolism Genes

**Figure 2:**
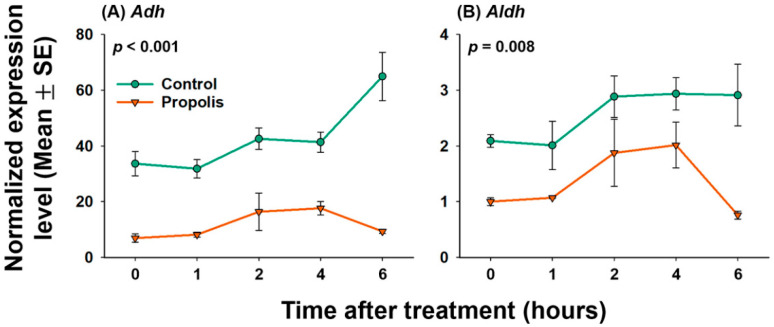
Expression of alcohol metabolism-related genes in propolis-fed *D. melanogaster* following ethanol exposure. Relative expression levels of *Adh* (**A**) and *Aldh* (**B**) in adult female *D. melanogaster* reared on a control or propolis-supplemented diet and exposed to 12.5% ethanol. Samples were collected at 0, 1, 2, 4, and 6 h after exposure. Gene expression was quantified by qRT-PCR and normalized to *RPL18*. Data are presented as mean ± SEM. Statistical analysis was performed using GLM repeated-measures ANOVA (*p* < 0.05).

To determine whether increased ethanol tolerance of propolis-treated flies was associated with altered ethanol metabolism, expression levels of *Adh* and *Aldh* were quantified following ethanol exposure. Across all time points examined, the expression levels of both genes were consistently lower in propolis-treated flies than in control flies (Figure 2).

In the control group, *Adh* expression increased progressively over time, rising from 33.64 ± 4.33 at 0 h to 81.22 ± 7.69 at 6 h. In contrast, propolis-treated flies exhibited relatively stable and substantially lower *Adh* expression, ranging from 6.90 ± 3.48 to 9.31 ± 0.77 over the same period (Figure 2A). Similarly, *Aldh* expression in propolis-treated flies displayed only modest fluctuations and remained significantly lower than that in the control group (Figure 2B). Statistical analysis confirmed significant treatment effects for both *Adh* (*p* < 0.001) and *Aldh* (*p* = 0.008) (Figure 2). These results indicate that enhanced ethanol tolerance in propolis-fed flies is not mediated by increased expression of classical ethanol-metabolizing enzymes.

### 3.3. Antioxidant-Related Gene Expression Is Not the Primary Driver of Propolis-Induced Ethanol Tolerance

**Figure 3:**
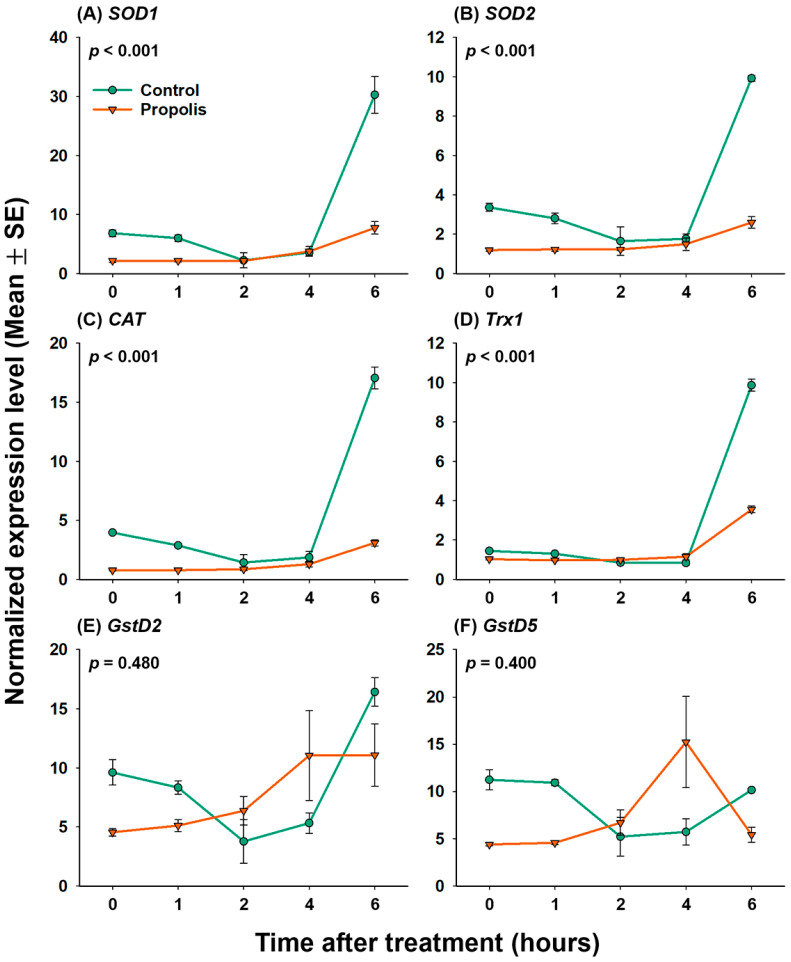
Expression of antioxidant-related genes in propolis-fed *D. melanogaster* following ethanol exposure. Relative expression levels of antioxidant-related genes, including *SOD1* (**A**), *SOD2* (**B**), *CAT* (**C**), *Trxr1* (**D**), *GstD2* (**E**), and *GstD5* (**F**), in adult female *D. melanogaster* reared on a control or propolis-supplemented diet and exposed to 12.5% ethanol. Samples were collected at 0, 1, 2, 4, and 6 h after exposure. Expression levels were normalized to *RPL18* and are presented as mean ± SEM. Statistical significance was assessed using GLM repeated measures ANOVA (*p* < 0.05).

We assessed whether propolis supplementation altered the transcriptional responses of antioxidant-related genes following ethanol exposure. Expression levels of *superoxide dismutase1* (*SOD1*), *superoxide dismutase2* (*SOD2*), *catalase* (*CAT*), *thioredoxin reductase-1* (*Trxr1*), *glutathione S transferase D2* (*GstD2*), and *glutathione S transferase D5* (*GstD5*) were analyzed across multiple time points (Figure 3).

During early exposure (0–1 h), propolis-treated flies exhibited lower expression of *SOD1*, *SOD2*, *CAT*, and *Trxr1* than control flies (Figure 3A–D). In the control group, expression of these genes declined gradually until 4 h, followed by a marked increase at 6 h. This late-stage induction was substantially attenuated in propolis-treated flies. For example, at 6 h, *SOD1* expression reached 38.8 ± 9.06 in control flies but only 7.28 ± 1.06 in propolis-treated flies, while *SOD2* reached 12.73 ± 2.81 and 2.59 ± 0.30, respectively (Figure 3A,B). *CAT* and *Trxr1* exhibited similar patterns, with significantly lower expression in propolis-treated flies at 6 h (Figure 3C,D). In contrast, *GstD2* and *GstD5* exhibited temporally variable expression patterns, but no significant treatment effects were detected (*GstD2*, *p* = 0.480; *GstD5*, *p* = 0.400) (Figure 3E,F).

Overall, although propolis supplementation modulated antioxidant-related gene expression, these changes did not correspond to a generalized upregulation of antioxidant defenses and are therefore unlikely to account directly for the enhanced ethanol tolerance observed.

### 3.4. Propolis Induces Sustained Upregulation of IMD Pathway-Dependent Antimicrobial Peptides

**Figure 4:**
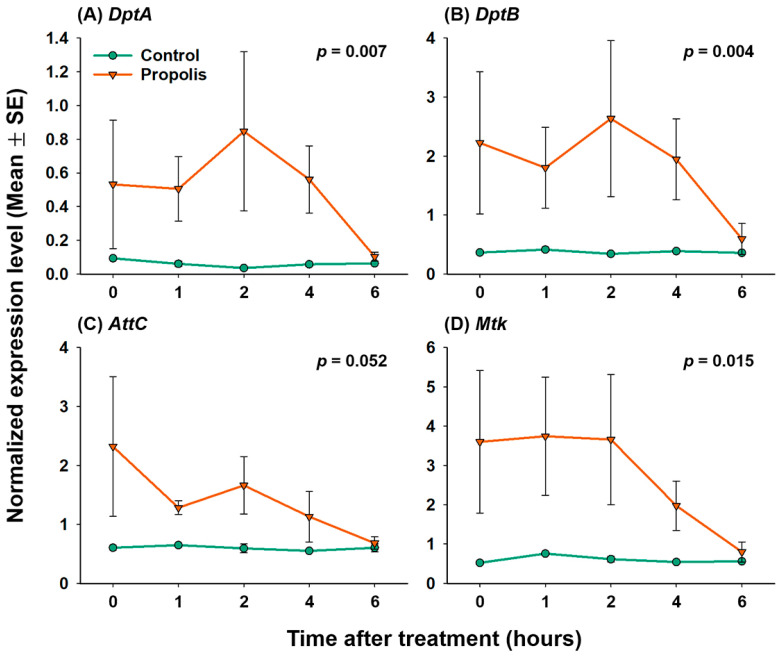
Upregulation of IMD-dependent antimicrobial peptide genes in propolis-fed *D. melanogaster*. Relative expression levels of antimicrobial peptide genes *DptA* (**A**), *DptB* (**B**), *AttC* (**C**), and *Mtk* (**D**) in adult female *D. melanogaster* reared on a control or propolis-supplemented diet and exposed to 12.5% ethanol. Samples were collected at 0, 1, 2, 4, and 6 h after exposure. Gene expression was quantified by qRT-PCR, normalized to *RPL18*, and expressed as mean ± SEM. Statistical analysis was performed using GLM repeated-measures ANOVA (*p* < 0.05).

In parallel with the survival assays, we examined whether dietary propolis supplementation influenced the expression of AMP genes associated with the IMD signaling pathway. Expression levels of *DptA*, *DptB*, *AttC*, and *Mtk* were quantified following ethanol exposure (Figure 4). Notably, propolis-treated flies exhibited elevated expression of all four AMP genes even before ethanol exposure (0 h), indicating a pre-existing immune-primed state. Following ethanol exposure, IMD-AMP expression remained consistently higher in propolis-treated flies than in control flies from 0 to 4 h, with convergence observed at 6 h.

*DptA* expression increased from 0.53 ± 0.38 at 0 h to 0.85 ± 0.47 at 2 h before gradually declining (Figure 4A). *DptB* and *AttC* displayed similar temporal dynamics, characterized by early elevation followed by gradual downregulation (Figure 4B,C). *Mtk* exhibited sustained expression through 2 h and declined thereafter (Figure 4D). In contrast, control flies maintained relatively low and stable IMD-AMP expression throughout the experimental period. Statistical analysis revealed significant treatment effects for *DptA* (*p* = 0.007), *DptB* (*p* = 0.004), and *Mtk* (*p* = 0.015), whereas *AttC* did not reach statistical significance (*p* = 0.052) (Figure 4).

These results demonstrate that lifelong dietary propolis supplementation induces sustained upregulation of IMD pathway-dependent AMPs and suggest that pre-emptive elevation of IMD-AMP expression is associated with enhanced ethanol tolerance.

## 4. Discussion

Insects are continuously exposed to diverse chemical stressors, and their survival depends on integrated physiological strategies that extend beyond classical detoxification pathways. In *D. melanogaster*, ethanol is a particularly important chemical stressor because this species predominantly inhabits decaying and fermenting fruits, where microbial activity generates ethanol at concentrations sufficient to induce physiological stress and oxidative damage [15]. Consistent with this ecological association, *D. melanogaster* exhibits strong ethanol preference and significantly higher ethanol tolerance than closely related species such as *D. suzukii*, which preferentially exploits fresh fruits with minimal fermentation [7,8,16]. These interspecific differences suggest that ethanol tolerance is a physiological specialization shaped by chronic exposure to fermentative environments.

Ethanol tolerance has traditionally been attributed to metabolic detoxification enzymes, particularly Adh and Aldh, as well as antioxidant-related enzymes (SOD, GST, CAT, and Trxr1) that mitigate ethanol-induced oxidative stress [1,2,17]. However, transcriptomic analyses of ethanol-exposed *D. melanogaster* have consistently revealed that innate immune genes, especially AMPs, are among the most strongly induced gene categories, whereas classical detoxification genes often exhibit limited or inconsistent responses [8]. These findings indicate that ethanol tolerance cannot be fully explained by canonical metabolic and antioxidant pathways alone. Our previous studies demonstrated that ethanol exposure robustly induces IMD pathway-dependent AMP genes and that RNAi-mediated suppression of the IMD pathway through *Relish* knockdown significantly reduces AMP expression and compromises survival under ethanol stress [9]. These results established that IMD-AMP induction is necessary for ethanol tolerance. However, because IMD-AMP upregulation in those studies occurred only after ethanol exposure, it was unclear whether IMD-AMPs functioned as active determinants of tolerance or merely reflected downstream responses to ethanol-induced oxidative stress or tissue damage.

This study addresses this unresolved question by demonstrating that pre-existing elevation of IMD-AMP expression before ethanol exposure enhances ethanol tolerance. Lifelong dietary supplementation with propolis increased baseline expression of IMD pathway-associated AMP genes before ethanol challenge, and this immune-primed state persisted during the early phases of exposure. Importantly, flies exhibiting increased IMD-AMP expression before ethanol exposure showed significantly improved survival, indicating that IMD-AMP upregulation can function as a proactive determinant of ethanol tolerance rather than merely a reactive consequence of chemical stress. By experimentally inducing IMD-AMP upregulation before chemical exposure, this study complements previous loss-of-function evidence and completes a causal framework in which IMD-dependent AMP expression is both necessary and, in a physiological context, sufficient to enhance ethanol tolerance in *D. melanogaster* [8,9].

From a physiological perspective, this temporal distinction between exposure-induced and pre-existing AMP expression is important. Ethanol exposure induces oxidative stress, disrupts epithelial integrity, and perturbs gut microbial homeostasis [15,18,19,20]. Elevated AMP levels before exposure may stabilize barrier tissues [21], limit secondary microbial proliferation [22], and facilitate rapid containment of stress-induced damage, thereby reducing the physiological burden during subsequent ethanol challenge [9]. In this context, AMPs appear to function not only as antimicrobial effectors but also as integrated stress-response molecules that enhance organismal resilience to ethanol-induced chemical stress, consistent with previous reports linking IMD pathway activation to chemical tolerance [9].

Importantly, enhanced ethanol tolerance in propolis-treated flies was not associated with increased expression of ethanol-metabolizing enzymes. Both *Adh* and *Aldh* expression levels were consistently lower in propolis-treated flies than in control flies. Excessive Adh activity can promote acetaldehyde accumulation and oxidative stress, which negatively affects survival and lifespan [1]. Thus, suppression rather than induction of ethanol-metabolizing enzymes may represent an adaptive strategy that limits the accumulation of toxic metabolic intermediates, further supporting the conclusion that propolis-induced ethanol tolerance operates independently of classical metabolic detoxification pathways.

Similarly, antioxidant-related gene expression did not exhibit patterns consistent with a primary role in propolis-induced ethanol tolerance. Although antioxidant enzymes such as SODs and CAT are essential components of oxidative stress defense [2], propolis-treated flies did not exhibit broad upregulation of these genes. In contrast, control flies displayed pronounced late-stage induction of antioxidant genes following prolonged ethanol exposure, likely reflecting compensatory responses to accumulated oxidative damage [15]. The attenuated antioxidant response observed in propolis-treated flies implies that bioactive compounds in propolis may reduce oxidative burden, thereby diminishing the requirement for strong endogenous antioxidant activation.

In contrast, IMD pathway-dependent AMP genes exhibited sustained upregulation in propolis-treated flies. Elevated IMD-AMP expression was evident even before ethanol exposure and remained higher than control levels during the early and intermediate phases of ethanol challenge, coinciding with the period of greatest survival advantage. These findings strongly support a proactive role for immune activation in ethanol tolerance, in which AMPs contribute directly to enhanced stress resilience rather than acting solely as downstream effectors of damage-induced signaling [23]. Together with our previous findings that RNAi-mediated suppression of IMD signaling reduced AMP expression and ethanol tolerance [9], the present results support a model in which propolis-induced IMD-AMP upregulation contributes to enhanced ethanol tolerance. The relatively large variation observed in IMD-AMP expression likely reflects the highly dynamic nature of immune activation during ethanol-induced stress. Because IMD-AMP genes are rapidly and transiently regulated through IMD-dependent signaling, individual differences in stress sensitivity, oxidative status, and physiological condition among surviving flies may contribute to increased transcriptional variability during ethanol exposure [10,24].

Propolis was used in this study not as a natural dietary component of *D. melanogaster* but as an experimental tool to induce sustained immune activation. Propolis is a chemically complex mixture rich in plant-derived polyphenols and flavonoids with well-documented antimicrobial and immunomodulatory properties [25]. In honeybees, propolis consumption modulates immune responses and increases AMP expression during immune challenge [11,26], supporting its suitability as a dietary immune-modulating agent for investigating AMP function in insects.

In conclusion, this study demonstrates that diet-induced upregulation of IMD pathway-dependent AMPs before chemical exposure is sufficient to enhance ethanol tolerance in *D. melanogaster*. By distinguishing exposure-induced immune activation from pre-existing IMD-AMP elevation, this study advances current understanding of immune–chemical stress interactions and highlights the importance of temporal dynamics in IMD-AMP regulation. More broadly, these findings identify IMD-AMPs as key effectors linking immune physiology to adaptation in chemically challenging environments and suggest that immune priming through dietary modulation is a general strategy for enhancing resilience to xenobiotic stress.

## Data Availability

The original contributions presented in this study are included in the article/Supplementary Material. Further inquiries can be directed to the corresponding author.

## Supplementary Materials

The following supporting information can be downloaded at: https://www.mdpi.com/article/10.3390/insects17060542/s1, Table S1. Primers used in qRT-PCR assay analysis in this study.
