# Supplementary material for: Pre-Emptive Upregulation of Antimicrobial Peptides by Dietary Propolis Improves Ethanol Tolerance in Drosophila melanogaster
Source: Insects. 2026 May 22;17(6):542. doi: 10.3390/insects17060542 (PMC13300170; doi:10.3390/insects17060542)
Supplement: Supplementary file 1 [file insects-17-00542-s001.zip › insects-4208411-supplementary.pdf]

Table S1. Primers used in qRT-PCR assay analysis in this study

Categories	Gene		Primers				Amplicons				Reference
	Full gene name	Symbol	Sequence (5‘ → 3')	Size (bp)	GC (%)	Tm (℃)	Size (bp)	GC (%)	R^2	Efficiency (%)	
Alcohol metabolism	*Alcohol dehydrogenase*	*Adh*_F	GCGAAACTGGCCCCCATTAC	20	60	62.5	175	62.3	0.996	92.1	This study
		*Adh*_R	CCTTGACGAAGTTCTCGGCG	20	60	62.5					
	Aldehyde dehydrogenase	*Aldh*_F	AGAAGCGCACCGTGGGCAAT	20	60	62.5	134	52.2	0.995	92.9	This study
		*Aldh*_R	GCCACCGGCAACCAACTTAG	20	60	62.5					
Antioxidant related	*Superoxide dismutase1*	*SOD1*_F	CCCACCAAGGTCAACATCACC	21	57.1	63.2	138	60.1	0.997	93.38	This study
		*SOD1*_R	GTTGCCCGTTGACTTGCTCAG	21	57.1	63.2					
	Superoxide dismutase2	*SOD2*_F	AAAGGAGCTGACCACGCTGAC	21	57.1	63.2	181	65.2	0.993	92.38	This study
		*SOD2*_R	CTGCAGATAGTAGGCGTGCTC	21	57.1	63.2					
	Catalase	*CAT*_F	CGGGATGGAGCCATGAATGTG	21	57.1	63.2	131	61	0.995	90.1	This study
		*CAT*_R	ACATCTCCAGTCACCGGACAG	21	57.1	63.2					
	Thioredoxin reductase-1	*Trxr1*_F	CAGTTCGGAGCCGATGAGATC	21	57.1	63.2	132	58.3	0.994	91.69	This study
		*Trxr1*_R	ATAGACGCGCTGGTCACCATG	21	57.1	63.2					
	Glutathione S transferase D2	*GstD2*_F	TCCACAGCACACCATTCCCAC	22	50	62.1	165	57	0.992	96.9	This study
		*GstD2*_R	CCCATGTCGAAGTACAGACGC	21	57.1	63.2					
	Glutathione S transferase D5	*GstD5*_F	TGGTGGAGAAGTACGGCAAGG	21	57.1	63.2	223	49.8	0.991	90.8	This study
		*GstD5*_R	TAGTTCTGGCCCTCCAGGAAG	21	57.1	63.2					

Table S1. Continued

Categories	Gene		Primers				Amplicons				Reference
	Full gene name	Symbol	Sequence (5‘ → 3')	Size (bp)	GC (%)	Tm (℃)	Size (bp)	GC (%)	R^2	Efficiency (%)	
Antimicrobial peptides	*Diptericin A*	*DptA*_F	TTACTTTGCTGCGCAATCGCTT	22	45	60.1	213	56.3	0.9915	90.73	[9]
		*DptA*_R	TCCATATCCTCCATTCAGTCCA	22	45	60.1					
	Diptericin B	*DptB*_F	GTGCGTCGCCAGTTCCAATTG	21	57	63.2	121	59.5	0.9967	92.62	[9]
		*DptB*_R	CATCGAAGGAGTGGCGTCCAT	21	57	63.2					
	Attacin-C	*AttC*_F	CCTCGCAGAATCAGCTTGCCA	21	57	63.2	136	51.5	0.9954	91.63	[9]
		*AttC*_R	CCCAGCTCCAATTGCTTACCC	21	57	63.2					
	Metchnikowin	*Mtk*_F	GCGATTTTTCTGGCCCTGCTG	21	57	63.2	115	68.3	0.9996	94	[9]
		*Mtk*_R	GGTTAGGATTGAAGGGCGACG	21	57	63.2					
Reference	*ribosomal protein L18*	*RPL 18*_F	GCAAGCCAGCACTGAATACG	20	55	60.1	197	51.8	0.9999	92.95	[14]
		*RPL 18*_R	TGCTGGCACTCAGGATGGTT	20	55	60.1					
